# Antitumor Effect of Lenvatinib Combined with Alisertib in Hepatocellular Carcinoma by Targeting the DNA Damage Pathway

**DOI:** 10.1155/2021/6613439

**Published:** 2021-07-22

**Authors:** Jianwen Hao, Qizhen Peng, Keruo Wang, Ge Yu, Yi Pan, Xiaoling Du, Na Hu, Xuening Zhang, Yu Qin, Huikai Li

**Affiliations:** ^1^Department of Radiology, Tianjin Chest Hospital, Tianjin 300350, China; ^2^Department of Radiology, Tungwah Hospital of Sun Yat-Sen University, Dongguan, 523000 Guangdong, China; ^3^Department of Diagnostics, Tianjin Medical University, Tianjin 300070, China; ^4^Department of Hepatobiliary, Tianjin Medical University Cancer Institute and Hospital, National Clinical Research Center for Cancer, Key Laboratory of Cancer Prevention and Therapy of Tianjin, Tianjin Clinical Research Center for Cancer, Tianjin 300070, China; ^5^Department of Pathology, Tianjin Medical University Cancer Institute and Hospital, National Clinical Research Center for Cancer, Key Laboratory of Cancer Prevention and Therapy of Tianjin, Tianjin Clinical Research Center for Cancer, Tianjin 300070, China; ^6^Department of Radiology Second Hospital of Tianjin Medical University, Tianjin 300070, China

## Abstract

**Methods:**

Immunohistochemical staining, sequencing, and genetic analysis of liver cancer tissues were performed. The antitumor efficacy of single-agent or combination treatment was measured by cell counting kit-8 assay and colony formation assays. Their antiproliferative and apoptosis activity is evaluated by cell cycle analyses and wound healing assays. The DNA-related proteins were also measured by Western blotting and immunohistochemical staining. The HepG2 xenograft model was used to detect the effects of lenvatinib-alisertib on the antitumor activity.

**Results:**

AURKA was found to be upregulated in HCC tissues (77.3%, 17/22). Combined alisertib and lenvatinib treatment significantly enhanced the inhibition of proliferation and migration in HepG2 and Hep3B cell lines compared to single-agent treatments (all *P*s < 0.01). Alisertib alone or in combination with lenvatinib demonstrated a significant increase in the percentage of super-G2 cells (lenvatinib 1 *μ*M vs. lenvatinib 1 *μ*M + alisertib 0.1 *μ*M 8.84 ± 0.84 vs. 34.0 ± 1.54, *P* < 0.001). Discontinuous spindles and missegregated chromosomes in HCC cells treated with alisertib in combination with lenvatinib were observed. We further revealed that combined treatment inhibited the expression of DNA damage pathway proteins compared to those of single-agent treatments. In nude mice, combined administration of alisertib combined with lenvatinib significantly enhanced the suppression of tumor growth and induced apoptosis (all *P*s < 0.01).

**Conclusions:**

Our findings provide evidence for the possible use of alisertib in combination with lenvatinib in the treatment of HCC for better therapeutic outcomes.

## 1. Introduction

Hepatocellular carcinoma (HCC), which constitutes 90% of primary liver malignancies, is deadly cancer with a rising global burden. Despite recent advances in diagnosis and treatment, poor prognosis, high recurrence, and rapid progression still put the advanced HCC a highly lethal disease [1, 2]. The current evidence-based HCC treatment involves sorafenib or lenvatinib being the first line, regorafenib or ramucirumab being the second line, and cabozantinib being the second- and third-line setting. A cumulative median overall survival (OS) of >20 months can be reached in patients with the maintained liver function [3]. In addition, Marasco et al. assessed the performance of several prognostic scores. They reported poor performance outcomes of HCC patients treated with sorafenib [4]. European Society for Medical Oncology (ESMO) guideline recommended considering all of these approved agents mentioned above in the second-line setting after atezolizumab-bevacizumab combination was more efficacious; median progression-free survival is only 6.8 months [3]. Chromosomal instability (CIN) is a major hallmark of hepatic tumorigenesis. It hampers prognosis and therapy, sensed as DNA damage, and induces a signaling pathway called the DNA damage response (DDR) [5]. Therefore, targeting DDR pathways is associated with a predisposition to cancer and affects responses to DNA-damaging anticancer therapy.

Aurora kinase A (AURKA), a serine/threonine kinase family member, is frequently overexpressed in HCC. Overexpression and amplification of AURKA in HCC have been associated with aggressive tumor characteristics, chemoresistance, and poor prognosis in HCC, indicating that AURKA plays a significant role in HCC [6]. Moreover, AURKA is a key cell cycle regulator critical for mitotic events and plays a key role upstream of CDK1, at the onset of mitosis and upon DNA damage [7, 8]. As a result, AURKA could be an attractive target for cancer therapy, and multiple inhibitors have been developed.

Alisertib (MLN8237), an investigational small molecule, is an orally effective selective AURKA inhibitor. Several clinical studies have reported the effectiveness of alisertib, such as advanced solid tumors, advanced malignancies, peritoneal carcinoma, head and neck squamous cell carcinoma, and advanced sarcomas [9]. Alisertib demonstrated the anticancer effect on various types of cancers in preclinical models. Besides, several phase I to III clinical trials investigating the effect of alisertib for advanced solid tumors and hematologic malignancies have been completed or are ongoing and have shown some promising results [9–11]. However, the evidence of the effect of alisertib on HCC is very limited. In addition, alisertib was found to be generally well tolerated with few mild to moderate side effects such as nausea, fatigue, and neutropenia [9, 12, 13].

The systematic treatment for HCC has dramatically changed over the past two years. As a tyrosine-protein kinase inhibitor (TKI), lenvatinib is a new orally administered, multikinase inhibitor that selectively inhibits VEGFR1–3, fibroblast growth factor receptor (FGFR) 1–4, PDGFR *α*, RET, and KIT [14] and has been used to treat progressive, locally recurrent or metastatic, radioactive iodine-refractory differentiated thyroid cancer (USA and EU). It has been approved for first-line treatment of unresectable HCC (uHCC) based on the phase III REFLECT study in China, USA, Japan, EU, and other areas this year [15]. Lenvatinib has been efficacious in patients with intermediate-stage HCC by reducing tumor size, who became TACE failures [16, 17]. It is generally well tolerated with manageable side events such as hypertension, diarrhea, loss of appetite, and weight [15]. In addition, REFLECT trial in patients with advanced HCC showed the potentiality of lenvatinib in advanced HCC treatment [18]. Nevertheless, immunotherapies, including immune checkpoint inhibitors, have had promising results in patients with advanced HCC, likely in part because of the contribution of both inflammation and suppressed immune microenvironments to the pathogenesis of HCC so that the lenvatinib combined with transarterial chemoembolization (TACE) and immune checkpoint inhibitors are currently on-going [19].

In the present study, we investigated the potential antitumor combined effect of alisertib-lenvatinib in HCC cells in vitro and xenograft tumors in vivo and determined the involved molecular mechanisms. We hypothesized that the alisertib-lenvatinib combination treatment would enhance cell cycle arrest, induce polyploidy and subsequent apoptosis in HCC cells, and trigger DNA damage pathway signaling.

## 2. Materials and Methods

### 2.1. Patient Samples and Cell Lines

A total of 22 of HCC specimens were obtained from HCC patients who were newly diagnosed at the Department of Hepatobiliary, Tianjin Medical University Cancer Institute and Hospital. The study was conducted in accordance with the Declaration of Helsinki and Good Clinical Practice guidelines. All patients have signed on the written informed consent. All specimens were cut into pieces of 40 mm cube and formalin fixed and paraffin embedded. The HepG2 and Hep3B cell lines were obtained from the American Type Culture Collection. They were cultured in DMEM (GIBCO, USA) supplemented with 10% fetal bovine serum (FBS), streptomycin (100 mg/mL), and penicillin (100 U/mL). All cells were cultured at 37°C in an atmosphere containing 5% CO_2_.

### 2.2. Targeted Next-Generation Sequencing and Genetic Analysis

Genomic profiling is an efficient method of screening mutations and effective in diagnosing genetic disease in the clinical setting, which was performed by Life Healthcare. According to the manufacturer's protocols, at least 100 ng of cancer tissue DNA was extracted from each 40 mm FFPE tumor sample using a DNA extraction kit (QIAamp DNA FFPE Tissue Kit). All coding exons of 601 key cancer-related genes and selected introns of 17 genes commonly rearranged in solid tumors were incorporated into the custom hybridization capture panel. In addition, the probe density was increased to ensure high efficiency of capture in the conservatively low-read depth region. Libraries were each diluted to 1.05 nmol/L and then sequenced with a mean coverage of 900× for FFPE samples and 300× for matched blood samples on an Illumina NextSeq-500 Platform (Illumina Incorporated).

### 2.3. Cell Screening Assay

The cell screening assay was used to test drug toxicity, safety, and efficacy. HepG2 and Hep3B cells morphological changes were detected by microscopy once the cells had been treated with alisertib and lenvatinib at different concentrations for 48 h at 37°C with DMSO.

### 2.4. Cell Viability and Cell Death

Cell viability was assessed using cell toxicity assays that indicate markers of cell death. Cells were seeded into 96-well plates at a density of 1 × 10^4^ cells per well in DMEM culture media with 10% FBS. Cell number was assessed by Cell Counting Kit-8 (MCE, USA) to determine cell viability. After incubation for 48 h, 10 *μ*l of CCK-8 reagent was added and measured at a wavelength of 450 nm.

### 2.5. Colony Formation Assay

The colony formation assay was done to examine the adhesion-independent cell proliferation of cancer cells. In this study, five hundred cells were seeded into each well of 6-well plates and cultured for 12 days. The cell colonies were fixed with precooled 4% paraformaldehyde for 30 min at room temperature. Then, the cells were subsequently stained with 0.5% crystal violet (Beyotime, China) for 30 minutes. After crystal violet staining, colony images were analyzed with ImageJ software. Independent experiments were conducted in triplicate.

### 2.6. Wound Healing Assay

The wound healing assay was used for studying cell migration and cell-cell interaction. Culture-Inserts (Ibidi, Germany) was used to measure cell migration. A cell suspension at a density of 7 × 10^4^/mL (70 *μ*L volume) was applied to each well of the culture inserts. After the appropriate duration for cell attachment (24 h), a cell-free gap of 500 *μ*M was created by removing the Culture-Insert. Images were captured every 24 h using an inverted phase-contrast microscope. The percent of wound closure in five randomly chosen fields was analyzed with ImageJ software.

### 2.7. Cell Cycle Analyses

Flow cytometry was used to distinguish cells in different phases of their cycle. The analysis of cell cycle progression was carried out by using a cell cycle analysis kit (Beyotime, China). Briefly, cells were harvested and incubated in 70% ethanol at 4°C overnight. Later, the cells were stained with 5 *μ*L propidium iodide (PI). After incubating away from light for 30 min, the samples were analyzed by a flow cytometer II (BD FACSCanto, San Jose, CA, USA). Data were analyzed by FlowJo software (Tree Star, Ashland, OR, USA).

### 2.8. Immunofluorescence Analyses

Immunofluorescence was used to detect antigens in cellular contexts utilizing antibodies. Cells seeded on glass coverslip in 12-well plates, fixed in 4% paraformaldehyde solution, and then blocked with immunol staining blocking buffer for 1 hour at room temperature and incubated with primary antibodies at 4°C overnight. This was followed by incubation with the fluorescent dye-conjugated secondary antibody Alexa Fluor 488 goat anti-rabbit or Alexa Fluor 555 donkey anti-mouse for 1 hour and stained with DAPI. Finally, images were taken under an inverted fluorescence microscope.

### 2.9. Foci Counting under Fluorescence Microscopy

Images were observed and captured manually by using a fluorescence microscope-DeltaVision (GE, USA). The numbers of green *γ*-H2AX foci in the nuclei were counted using Apache Velocity Project software. Overall, 50-100 cells were obtained for each specimen to calculate the number of foci per cell.

### 2.10. TUNEL Assay

The TUNEL assay was used for detecting apoptotic DNA fragmentation, apoptotic cells, or cellular DNA breakage. Apoptosis in transplanted tumor tissues was detected using a TUNEL assay and performed according to the guidelines recommended by the TUNEL assay kit (Roche, Germany).

### 2.11. Mouse Xenograft Model

The mouse xenograft model was conducted to test anticancer therapies and researches. All animal experiments strictly followed the guidelines of the Institute of Hematology, Chinese Academy of Medical Science. Approximately 5.0 × 10^6^ HepG2 cells were suspended in 100 *μ*L of PBS and injected subcutaneously into the right side of the posterior flank of female BALB/c athymic nude mice (Institute of Hematology, Chinese Academy of Medical Science, 5-6 weeks). Tumor volume was calculated as follows: tumor volume = length × width^2^/2. When the average tumor size reached approximately 50 mm^3^, alisertib, lenvatinib, or the combination of alisertib and lenvatinib was administered via intraperitoneal injection, 30 mg/kg every 3 days, for 4 consecutive weeks. After 4 weeks, all mice were killed, and necropsies were performed. The primary tumor tissue was stained and analyzed by hematoxylin and eosin (H&E) and TUNEL staining.

### 2.12. Western Blotting

Western blotting was conducted to detect the protein expression of DNA damage signaling pathways. Cells pellets were lysed by RIPA lysis buffer. Then, 20-40 *μ*g protein from each experimental condition was subjected to sodium dodecyl sulfate-polyacrylamide gel for electrophoresis, then transferred onto a polyvinylidene fluoride membrane. The membranes were blocked with QuickBlock™ Primary Antibody Dilution Buffer (Beyotime, China) for 10 min at room temperature and then incubated with the appropriate primary antibody overnight at 4°C. Primary antibodies were diluted in Primary Antibody Dilution Buffer (Beyotime, China). The membranes were washed with TBS-T, followed by probing with species-specific secondary antibodies conjugated with HRP, diluted in Secondary Antibody Dilution Buffer (Beyotime, China). Protein bands were detected using Enhanced Chemiluminescence Substrate (PerkinElmer, USA).

### 2.13. Antibodies and Reagents

The antibodies used in this study were as follows: anti-Aurora A antibody, Abcam, #ab52973; DNA Damage Antibody Sampler Kit, CST, #9947; mouse anti-alpha tubulin antibody, Abcam, #7291; donkey anti-mouse IgG (H + L) highly cross-adsorbed secondary antibody, Alexa Fluor 555, Invitrogen, #A-31570; and Alexa Fluor 488-labeled goat anti-rabbit IgG (H + L), Beyotime, #A0423. Lenvatinib (E7080) and alisertib (MLN8237) were purchased from Selleck.

### 2.14. Statistical Analysis

All statistical analyses were conducted using GraphPad Prism 8 biostatistics software (GraphPad Software, Inc., CA, USA). The experimental data are presented as the means ± standard error of the mean. Two-group comparisons were performed with Student's *t*-test. Multiple group comparisons were analyzed with one-way ANOVA. All tests performed were two-sided. *P* < 0.05 was considered statistically significant.

## 3. Results

### 3.1. AURKA Was Upregulated in Primary Liver Cancer Tissues

**Figure 1:**
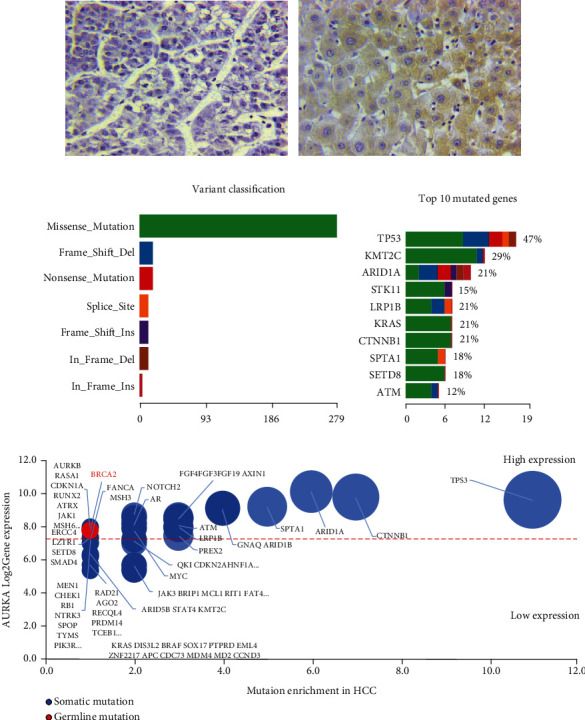
AURKA was upregulated in HCC tissues. (a) Immunohistochemistry staining for AURKA in HCC tissues and ChC tissues. (b) A colored bar plot displays the number of variant classifications. (c) Variants of per-sample mutation burden. The stacked bar plot shows the variants of each sample in the HCC groups according to mutation classification. (d) Stacked bar plot depicting the variant types of the top 10 mutated genes sorted by decreasing frequency in the HCC groups. (e) Scatterplot of DNA mutation enrichment (*x*-axis) and gene expression (*y*-axis) in hepatic carcinoma and cholangiocarcinoma patients. The enrichment (dashed line) level is represented as the difference in mutation gene enrichment frequency between the two indicated gene expression levels. The *y*-axis shows the log2 AURKA expression for each mutated gene obtained from the intensity of AURKA and the range of AURKA. The red color represents germline mutation, and the blue color represents somatic mutation.

In this study, similar to other studies, the expression of AURKA was significantly upregulated (77.3%, 17/22) in 22 pathologically confirmed HCC patient's samples (Figure 1(a)). By analyzed targeted next-generation sequencing panel captured mutations in coding regions, we found the correlation between the expression of AURKA and mutations in 450 cancer-related genes in HCC samples. A missense mutation was the most common mutational type with low rates in other mutation variant classifications (Figure 1(b)). Among them, TP53 mutations were detected in 47% of the samples, along with 2 genes, KMT2C and ARID1A with greater than 20% mutation rates, and 7 other genes, STK11, LRP1B, KRAS, CTNNB1, SPTA1, SETD8, and ATM with over 10% mutation rates, including (Figure 1(c)). For mutation enrichment analysis, TP53 showed a higher AURKA significance correlation in HCC, indicating that the AURKA overexpression is correlated with TP53 mutations in HCC (Figure 1(d)).

### 3.2. Alisertib Significantly Enhances the Cytotoxic and Antimetastatic Activity of Lenvatinib in HCC Cells

**Figure 2:**
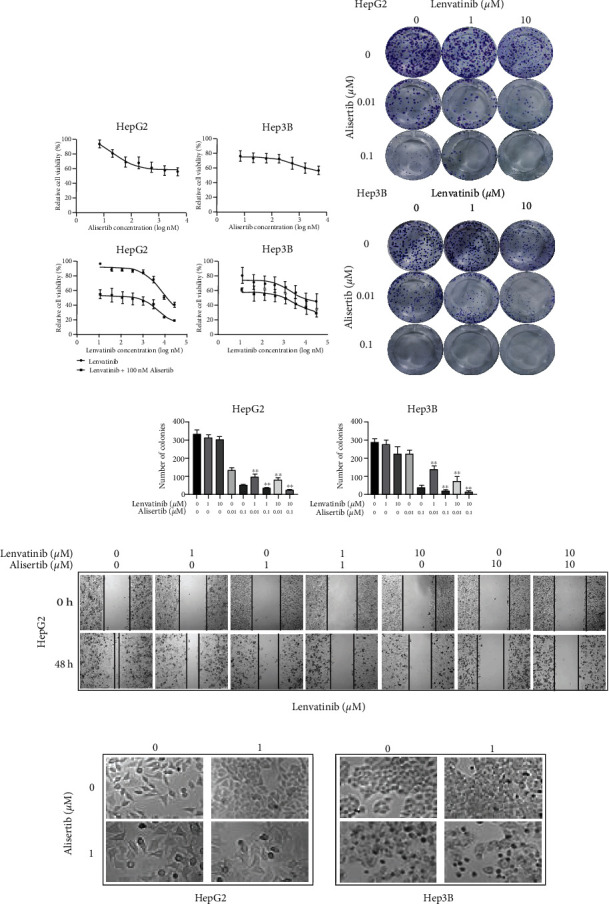
Alisertib significantly enhances the cytotoxic effect and antimetastatic activity of lenvatinib in HCC cells. (a) The cytotoxicity of various concentrations of alisertib and lenvatinib on HepG2 and Hep3B cells when administered alone or in combination. (b) Clonogenicity of HepG2 and Hep3B cells treated with alisertib alone, lenvatinib alone, and in combination. The results (from three independent experiments) were subjected to statistical analysis and are summarized in (c). (d) HepG2 and PLC cells were treated with lenvatinib, alisertib, or both for 24 or 48 hr. A wound-healing assay was then conducted to examine the invasive and metastatic capabilities of HepG2 and PLC cells. The results (from three independent experiments) were subjected to statistical analysis and are summarized in (e). (f) HepG2 and Hep3B cells were treated with lenvatinib, alisertib, or both for 48 hr. Morphological changes in the cells were detected by microscopy (magnification, ×20; scale bars, 50 *μ*m).

To elucidate the effect of alisertib on the cytotoxicity of HCC, treated HepG2 and Hep3B cells with various concentrations of alisertib (7–5000 nM) for 48 hours and concentration-survival curves were plotted. The results clearly showed that alisertib inhibited cell growth in a concentration-dependent manner (Figure 2(a)) and cotreated with alisertib significantly enhanced the cytotoxicity of lenvatinib. Furthermore, similar results showed that the combination of lenvatinib and alisertib exerted a more significant antiproliferative effect in HepG2 and Hep3B cells (Figures 2(b) and 2(c)). In the scratch wound healing assay, compared with alisertib or lenvatinib alone, the migratory capabilities in cell line HepG2 were also significantly reduced with the combination of alisertib and lenvatinib treatment (Figure 2(d)). In addition, marked morphological changes, such as enlarged, rounded, and swollen cells or detached and shrunken cells, were also noted following alisertib and/or lenvatinib (Figure 2(e)). All the above results demonstrate that alisertib enhances the cytotoxic and antimetastatic effects of lenvatinib in HCC cells by inhibiting proliferation and migration.

### 3.3. Alisertib Enhances the Antiproliferative Activity of Lenvatinib in HCC Cells

**Figure 3:**
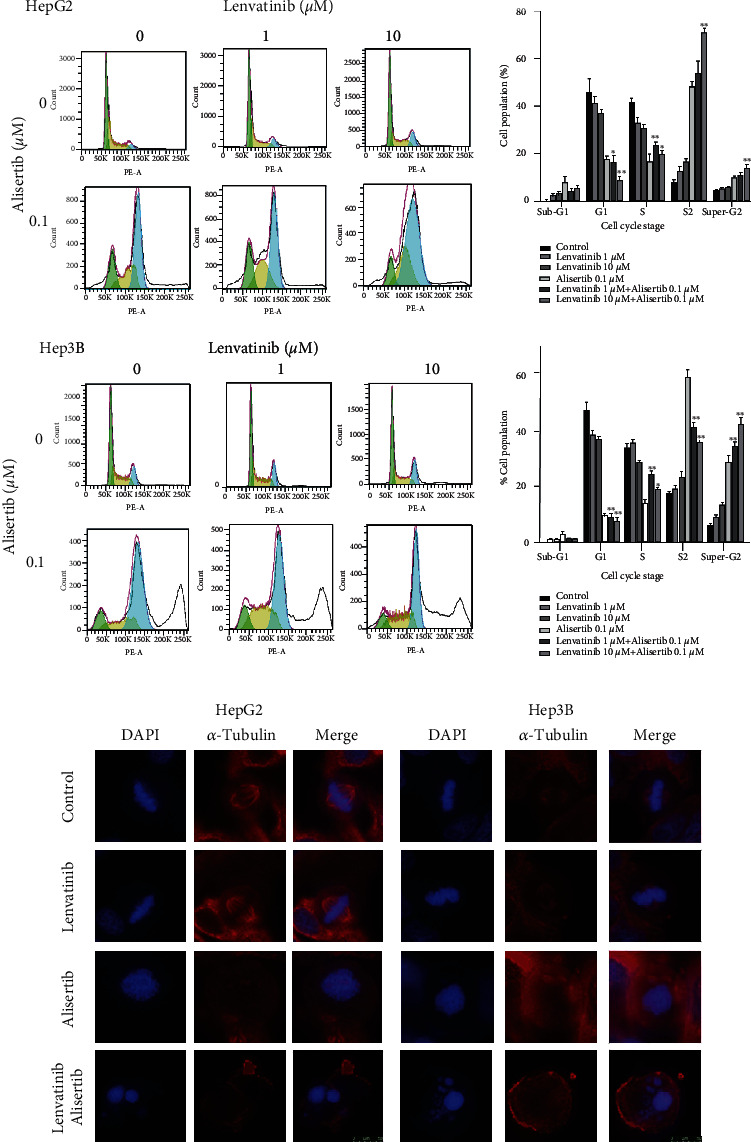
Alisertib enhances the antiproliferative activity of lenvatinib in HCC cells by blocking cell cycle progression and increasing apoptosis. (a) HepG2 and Hep3B cells were treated with lenvatinib, alisertib, or both for 48 hr. Flow cytometric analysis was then conducted to evaluate the cell cycle, and statistical analysis was performed. (b) HepG2 and Hep3B cells were treated with lenvatinib, alisertib, or both for 48 hr. Immunofluorescent staining of the spindles in cells used an antibody against *α*-tubulin (depicted in red). DNA was counterstained with DAPI (depicted in blue).

To explore the combined effects of alisertib and lenvatinib on the cell cycle of tumor cells in flow cytometric analysis, it revealed that alisertib and/or lenvatinib markedly reduced the percentage of G1 cells and significantly delayed the G2-M phase transition in HepG2 and Hep3B cells. Moreover, the alisertib alone or in combination with lenvatinib induced a significant increase in the percentage of super-G2 cells, which was accompanied by a drastic reduction in S-phase cell numbers (Figure 3(a)). The above results indicated that combined treatment of alisertib and lenvatinib induced tumor cell death by delaying the G2-M phase transition, reducing the S phase cell number and activating proapoptotic mechanisms. In addition, immunofluorescent staining of *α*-tubulin in confocal microscopy, discontinuous spindles, and missegregated chromosomes in HepG2 cells treated with alisertib in combination with lenvatinib was observed.

In contrast, cells treated with lenvatinib alone had more nicely ordered chromosomes in metaphase (Figure 3(b)), which showed that lenvatinib alone had little effect on the apoptosis of HCC cells. However, the apoptosis-promoting effect was more pronounced when lenvatinib was administered in combination with alisertib. All above results support our hypothesis that alisertib enhances the antitumor effect of lenvatinib by blocking cell cycle progression and enhancing apoptosis.

### 3.4. Alisertib Enhances the Inhibition of Lenvatinib in the DNA Damage Signaling Pathways

**Figure 4:**
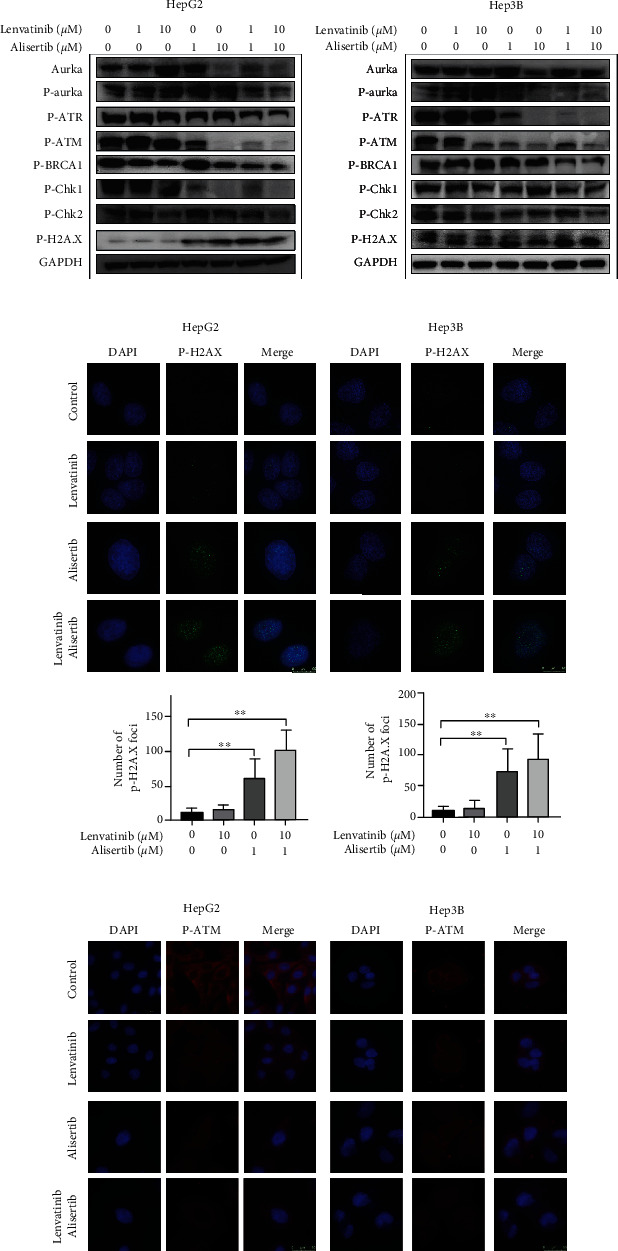
Lenvatinib-alisertib combination inhibits DNA damage signaling pathways in HCC cells. (a) HepG2 and Hep3B cells were treated with lenvatinib, alisertib, or both for 48 hr. Western blotting was then conducted to monitor the expression of AURKA, p-AURKA, p-ATR, p-ATM, p-BRCA1, p-Chk1, p-Chk2, and *γ*-H2AX in the cells. (b, c) HepG2 and Hep3B cells were treated with lenvatinib, alisertib, or both for 48 hr. Immunofluorescent staining of *γ*-H2AX or p-ATM and confocal microscopy analysis of the number of *γ*-H2AX foci was performed.

Subsequently, we explored the potential molecular mechanisms of the alisertib and lenvatinib combination in induces antitumor activity in HCC cells. Alisertib and lenvatinib combined treatment markedly reduced AURKA and p-AURKA protein expression levels both in HepG2 and Hep3B cells compared with that of either of them treated alone. Virtually, no decrease was observed in p-ATR and p-ATM protein levels after treatment with 1 *μ*M lenvatinib alone in HepG2 cells. However, when increased the concentration of lenvatinib to 10 *μ*M, the p-ATM expression was reduced along with p-Chk2. In contrast, alisertib alone inhibited the expression of DNA damage pathway proteins, such as p-ATR, p-ATM, p-BRCA1, p-Chk1, and p-Chk2. More importantly, the combination of lenvatinib and alisertib significantly inhibited the protein expression of p-ATR, p-ATM, p-BRCA1, p-Chk1, and p-Chk2 in both HepG2 and Hep3B cells. The low expression of the *γ*-H2AX protein was observed with lenvatinib alone. At the same time, alisertib increased the expression of the *γ*-H2AX protein. The combination of lenvatinib and alisertib markedly increased the expression of the *γ*-H2AX protein in HepG2 and Hep3B cells (Figure 4(a)). More interestingly, results in immunofluorescent staining of *γ*-H2AX or p-ATM and confocal microscopy analysis were consistent with the Western blotting (Figures 4(b) and 4(c)). All results suggest that alisertib enhances the antitumor effect of lenvatinib by inhibiting the DNA damage signaling pathways in HCC cells.

### 3.5. The Combination of Alisertib-Lenvatinib Enhances the Antiproliferative and Proapoptotic Activities In Vivo

**Figure 5:**
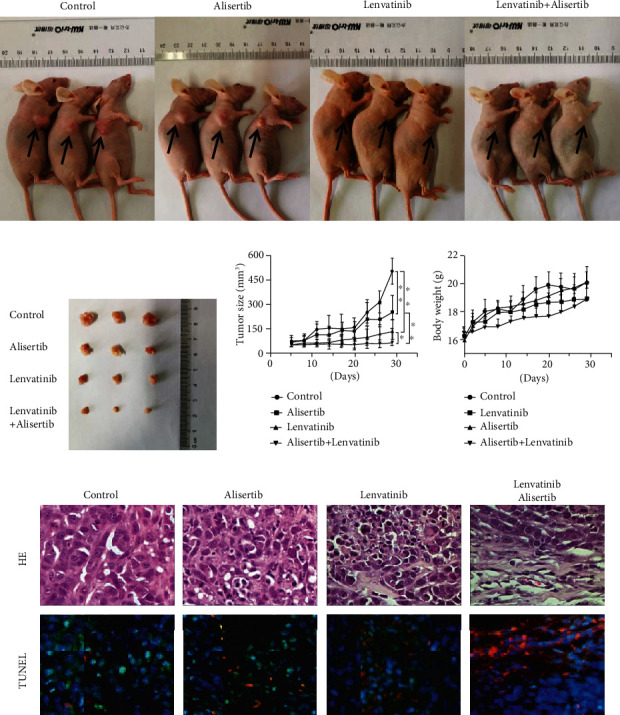
Alisertib significantly enhances the antiproliferative and proapoptotic activities of lenvatinib in HCC cells in vivo. Images of control, alisertib-, lenvatinib- and alisertib-lenvatinib combination-treated tumor-bearing mice. (b) Images of subcutaneous xenograft tumors. (c) Changes in mouse body weights during the formation of subcutaneous xenograft tumors. (d) The growth curves of the subcutaneous xenograft tumors. (e) Representative images of H&E staining and TUNEL staining. ^∗∗^*P* < 0.01 and ^∗∗∗^*P* < 0.001. Data are expressed as the mean ± SD. All experiments were repeated at least three times.

We assessed the in vivo antitumor efficacy of alisertib combined with lenvatinib in nude mice bearing Hep3B xenografts. Mice were randomly assigned to four groups: control, alisertib, lenvatinib, and combination treatment. Compared with the control condition, the oral administration of alisertib or lenvatinib resulted in significant tumor growth inhibition. Combining alisertib and lenvatinib significantly increased tumor growth suppression compared with single drugs alone (Figures 5(a)–5(c)). Either treatment had no significant effects on body weight in the nude mice (Figure 5(d)). In fluorescence microscopy and a TUNEL assay, it also showed that combined treatment with alisertib and lenvatinib significantly enhanced tumor apoptosis in mice (Figure 5(e), the nuclei: stained blue, the apoptotic cells: stained red, and the viable cell: stained green).

## 4. Discussion

This study is aimed at examining the inhibition of HCC cells by the combination of alisertib and lenvatinib. We found that the drug combination suppressed the cell proliferation and enhanced the apoptosis in vitro and in vivo. Alisertib enhances the antitumor effect of lenvatinib by inhibiting the DNA damage signaling pathways in HCC cells.

Lenvatinib has become an indispensable part of treatment regimens for patients with advanced hepatocellular carcinoma, with an unmet need for TKI therapy [16, 17, 20–25]. Preclinical studies demonstrated that lenvatinib has potent antiangiogenic activity by inhibited both the VEGF and FGF signaling pathways. It shows antitumor activity consistently across diverse solid tumor models, such as thyroid cancer, renal cell carcinoma, and HCC [26–28]. However, some real-world studies showed that the baseline characteristics, changes in serum biomarkers, and gene sequencing might hold the key for lenvatinib responses. As an AURKA inhibitor, alisertib has been used singly or combined treatment for advanced solid tumors and hematological malignancies in phase I, II, and III studies [12, 13, 29–33]. Although in several studies, AURKA inhibitors showed antitumor activity in combination with other chemotherapeutic agents. The evidence of the alisertib combined with lenvatinib in HCC models is still very limited. Our current study first suggested a potential therapeutic benefit of combined alisertib and lenvatinib in HCC treatment.

The TP53 gene is one of the most frequently mutated genes in HCC and other human cancers. Cancer cells with TP53 mutants generally acquire numerous characteristic alterations that may facilitate their oncogenic growth, chemoresistance, and metastasis [34–39]. Its mutation often indicates a poor prognosis for patients with HCC [40]. TP53 mutation leads to overexpression of AURKA in prostatic small cell neuroendocrine carcinoma [41]. The AURKA overexpression was observed in many tumors, and AURKA promoted the oncogenic effects of c-Myc, which is frequently amplified and overexpressed in HCC and many other cancers [42–45]. In addition, mutant p53 can target the VEGFR2 promoter transcriptional start site and plays a role in maintaining an open conformation at that location [46] and also served as predictive biomarkers for the response of VEGFR inhibitor in advanced sarcomas [47].

Interestingly, we utilized p53-deletion (Hep3B) and wild-type (HepG2) cell lines and obtained similar results. These findings suggested that AURKA-targeted therapy alone and in combination with lenvatinib are effective independent of p53 status. Here, based on the highly significant correlation between TP53 mutation and AURKA expression in HCC tissue, the alisertib and lenvatinib combination contributes to the anticancer activity against HCC, caused by combination suppressed cell survival in vitro and tumor growth in vivo.

**Figure 6:**
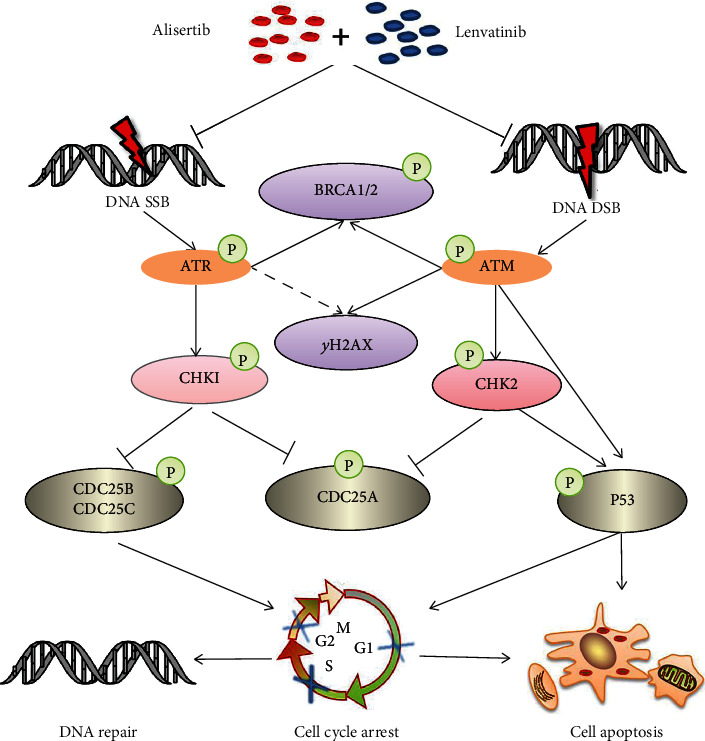
Schematic representation of the antitumor mechanism of the lenvatinib-alisertib combination in HCC. Alisertib, a novel AURKA inhibitor that affects the DNA damage pathway and its downstream signaling, induces hepatocellular carcinoma cell death together with lenvatinib.

On the other way, DNA is constantly damaged by a large variety of endogenous and exogenous influences. There is two cores in DNA damage signaling apparatus, a pair of related protein kinases, ATM (ataxia telangiectasia mutated) and ATR (ATM and Rad3-related), with their two downstream kinases, checkpoint kinases 1 and 2 (Chk1 and Chk2) [48, 49]. The ATM/CHK2 and ATR/CHK1 pathways cooperated in supporting genomic stability by modified cell cycle progression with DNA repair and controlled cell cycle transitions, DNA replication, and apoptosis [49]. The present study confirmed that the alisertib-lenvatinib combination enhanced its antitumor effects by inhibiting the DNA damage signaling pathway. As summarized in Figure 6, alisertib combined with lenvatinib reduced phospho-ATM to a much higher degree, directly or indirectly, through the CHK2 activation and phosphorylated in cell cycle blocking and apoptosis. Moreover, the combination treatment also affected ATR/CHK1. It led to a modified intra-S-phase cell cycle checkpoint in S-phase progression and response to DNA damage.

This study has several limitations, which are as follows: (i) this study did not investigate whether the alisertib-lenvatinib combination effect is “additive” or “synergistic.” (ii) For the efficacy in the treatment of advanced HCC, comparisons of alisertib-lenvatinib combination with other promising combination(s) such as lenvatinib-immune checkpoint inhibitor (i.e., pembrolizumab) have not performed in this study. (iii) Follow-up investigation via repeated infusion of alisertib-lenvatinib combination to assess their effect over time has not been performed. Thus, we plan to investigate and overcome these limitations in our future research on this topic with extended experiments.

To sum up, this study is one of the initial investigations of this topic. Thus, (i) comparisons between two or more combinations should be performed to find better efficacious and tolerable drugs in future studies. (ii) According to the statement of the ESMO guideline [4], all the available first- and second-line drugs for HCC should be treated in a second-line setting. This means that along with atezolizumab-bevacizumab or alisertib-lenvatinib combinations, more combinations such as lenvatinib-pembrolizumab should be investigated for more first-line setting. (iii) The “additive” or “synergistic” effect of these combinations such as alisertib-lenvatinib should be investigated to find out more efficacious combinations in future studies.

## 5. Conclusion

Our study demonstrated that the alisertib-lenvatinib combination contributes to the anti-HCC activity. They work together to inhibit the activation of DNA damage signaling pathways, thereby affecting various biological behaviors in HCC. Our findings provide evidence that AURKA inhibitor combination with lenvatinib would be a therapeutic approach for the treatment of advanced HCC.

## Data Availability

The underlying data could be found by our email: tjchlhk@126.com
